# Multi-Scale Structure–Mechanical Property Relations of Graphene-Based Layer Materials

**DOI:** 10.3390/ma14164757

**Published:** 2021-08-23

**Authors:** Jingran Liu, Huasong Qin, Yilun Liu

**Affiliations:** 1 Laboratory for Multi-Scale Mechanics and Medical Science, SV LAB, School of Aerospace, Xi’an Jiaotong University, Xi’an 710049, China; liujingran@stu.xjtu.edu.cn; 2 Key Laboratory for Intelligent Nano Materials and Devices of the Ministry of Education, Nanjing University of Aeronautics and Astronautics, Nanjing 210016, China

**Keywords:** graphene-based layer materials, multi-scale structure–property relations, mechanical behaviors, hierarchical structures, multi-scale optimization

## Abstract

Pristine graphene is one of the strongest materials known in the world, and may play important roles in structural and functional materials. In order to utilize the extraordinary mechanical properties in practical engineering structures, graphene should be assembled into macroscopic structures such as graphene-based papers, fibers, foams, etc. However, the mechanical properties of graphene-based materials such as Young’s modulus and strength are 1–2 orders lower than those of pristine monolayer graphene. Many efforts have been made to unveil the multi-scale structure–property relations of graphene-based materials with hierarchical structures spanning the nanoscale to macroscale, and significant achievements have been obtained to improve the mechanical performance of graphene-based materials through composition and structure optimization across multi-scale. This review aims at summarizing the currently theoretical, simulation, and experimental efforts devoted to the multi-scale structure–property relation of graphene-based layer materials including defective monolayer graphene, nacre-like and laminar nanostructures of multilayer graphene, graphene-based papers, fibers, aerogels, and graphene/polymer composites. The mechanisms of mechanical property degradation across the multi-scale are discussed, based on which some multi-scale optimization strategies are presented to further improve the mechanical properties of graphene-based layer materials. We expect that this review can provide useful insights into the continuous improvement of mechanical properties of graphene-based layer materials.

## 1. Introduction

**Figure 1:**
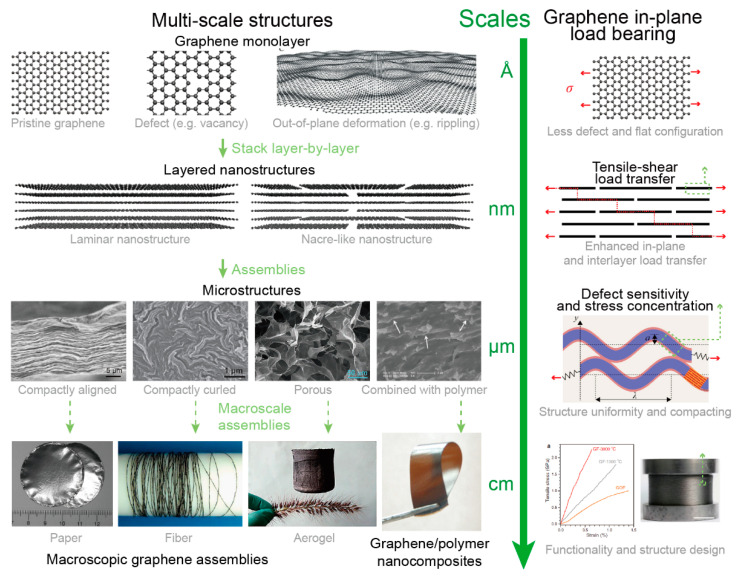
Multi-scale structures of graphene-based materials and the key loading bearing structures in each scale. Reprinted with permission from [8,9,10,11,12,13,14].

Graphene [1] has triggered great interest in two-dimensional (2D) materials in the past two decades. As the first discovered 2D material, graphene is the thinnest, strongest, and most flexible material known to exist. Accompanied by its large specific surface area, excellent thermal [2] and electrical [3] conductivities, graphene has been regarded as the ideal material for various advanced applications including flexible electronics [4], sensors [5,6], biomedicine [7], etc. In order to utilize the extraordinary mechanical properties of graphene in practical engineering structures, graphene is usually assembled into macroscopic structures. However, among the existing works, the mechanical properties have decreased about two orders from monolayer graphene to macroscopic graphene assemblies such as graphene-based papers and fibers, indicating multi-scale mechanical degradation mechanisms from the nanoscale to millimeter scale. Thus, bringing the excellent mechanical properties of monolayer graphene into its macroscopic assemblies across different length scales is extremely important for the practical applications of graphene-based materials and has attracted wide research interests, as shown in Figure 1. Many theoretical, simulation, and experimental works have been conducted to unveil the multi-scale structure–property relations of graphene-based materials for further improving their mechanical properties.

Monolayer graphene is the smallest building unit of graphene-based materials. In general, graphene can be fabricated by two approaches, that is “top-down” (i.e., mechanical cleavage [1,15], liquid exfoliation [16], and ion intercalation [17]) and “bottom-up” (i.e., chemical vapor deposition (CVD) [18] and wet chemical synthesis [19]). In these fabricating processes, defects are inevitably introduced, which cannot be ignored in determining the mechanical properties of graphene. On the other hand, the sixfold symmetry of graphene indicates isotropic mechanical properties in the basal plane, but under finite deformation, the mechanical properties of graphene are anisotropic. Therefore, some works have focused on revealing the structure–property relations of monolayer graphene. For example, continuum models have been developed to predict the elastic and failure behaviors of monolayer graphene mediated by defects. Furthermore, more attention has been played to investigate the mechanical behaviors of defective graphene based on molecule dynamic (MD) simulations. In general, defect is one of the major sources of the mechanical degradation of monolayer graphene. However, for some special cases, defects can increase the failure strain, fracture toughness, and out-of-plane deformation. Counterintuitively, in experiments, defects have been found to increase the Young’s modulus to almost twice with a vacancy content of ~0.2% [20]. Up until now, it is still a big challenge to accurately control the type and distribution of defects in monolayer graphene as well as to predict the mechanical properties of graphene with arbitrary defects.

Graphene layered nanostructures (GLNs)—the nanoscale building blocks of graphene-based materials—can be formed by assembling monolayer graphene in a layer-by-layer manner. According to the continuity of the graphene sheet in GLNs, we classify GLNs into two types, that is, the nacre-like structure and laminar structure. In general, the in-plane mechanical behaviors of the nacre-like structure as well as out-of-plane mechanical behaviors of the laminar structure have been studied. For the nacre-like structure subjected to in-plane tensile load, the in-plane stress in one graphene sheet is transferred to the adjacent graphene sheet via interlayer shearing. Related continuum models [1,21,22,23] have implied that in-plane tensile behaviors of this kind of structure mostly depend on the balance of in-plane and interlayer load transfer. In particular, the interlayer interaction of pristine graphene is van der Waals (vdW) interaction, too weak to transfer enough in-plane tensile load, which significantly limits the improvement in the mechanical properties of GLNs. Therefore, many strategies have been proposed to enhance the interlayer load transfer including chemical crosslinking [24,25,26,27] and nano-structural modifications. On the other hand, the out-of-plane behaviors of laminar structures including bending deformation [28,29,30,31], failure [32], and compressive buckling [33,34,35] have been studied. Due to the weak interlayer interactions, laminar structures have shown some unusual mechanical behaviors such as intrinsic buckling without structure slenderness. Although it is known the interlayer mechanical properties play an important role in the overall mechanical behaviors of both nacre-like and laminar structures, few works have been made to explore the effects of interlayer interaction on bending behaviors of laminar structures. Furthermore, the bending behaviors of nacre-like structures are still unknown.

Further assembly of GLNs can form the graphene-based layer materials (GLMs), whose mechanical properties dependent on not only the properties of GLNs, but also multi-scale assembling structures such as the wrinkles, orientation of GLNs, etc. Three kinds of macroscopic graphene assemblies, namely graphene papers (GPs) [11,36], graphene fibers (GFs) [12,37], and graphene aerogels (GAs) [38,39] have mostly been studied. The GPs and GFs are obtained with the GLNs assembled in a compacted manner, while the GAs possess cellular structures, but their cell wall consists of GLNs. Different methods have been developed to fabricate the different graphene macroscopic assemblies. For example, the compacted paper-like structures can be obtained from the vacuum filtration, while the compacted fiber-like structures come out of the wet-spinning method, and the porous structures result from the solution assembling, followed by freeze-dry methods. The details of different fabrication methods are briefly discussed in Section 4. Moreover, some porous films or fibers [40,41], exhibiting paper or fiber-like morphologies but possessing the porous structures inside, are produced via the combination of the fabrication methods of compacted and porous structures. In general, the structure compaction and uniformity are two important parameters to influence the load-bearing properties of GLMs in experiments. This is why GFs have superior mechanical properties, especially Young’s modulus and tensile strength, to that of GPs. However, the multi-scale mechanisms of mechanical property degradation from monolayer graphene to macroscopic graphene assemblies as well as the structure–property relations of GLMs are still unclear.

Various graphene/polymer nanocomposites have been developed. Thanks to its excellent mechanical properties, graphene is a promising reinforced filler that can significantly enhance the Young’s modulus and strength of composites with only a small amount of graphene. However, the uniform dispersion of graphene in composites with a large graphene content is a large problem, and the weak vdW interface between graphene and polymer matrix also provides the crack initiation source, which hinders the further mechanical improvement in graphene/polymer composites. Therefore, several strategies have been proposed to optimize the interface structures and properties to improve the overall mechanical properties of graphene nanocomposites including Young’s modulus, strength, and toughness [42,43]. Besides, other structures such as inverse nacre-like [44,45] or bi-continuous [46] laminar microstructures have been fabricated, which can simultaneously improve the strength and toughness of graphene nanocomposites.

Although several reviews have focused on graphene monolayer [47,48,49], GPs [50,51], GFs [52,53], GAs [54], and graphene/polymer nanocomposites [55,56], the mechanical degradation mechanisms of GLMs across a multi-scale have rarely been discussed. With this scenario, this review aims to provide an overall understanding of the structure–mechanical property relations of graphene-based materials from the nanoscale to macroscale to provide some multi-scale optimization strategies to improve their mechanical properties. We primarily focus on the hierarchical structures as well as the related mechanical behaviors of GLMs, accompanied by a brief introduction of the fabricating methods resulting in such microstructures. The rest of this review is organized as follows. In Section 2, theoretical models for monolayer graphene, nacre-like, and laminar GLNs are discussed. Note that the theoretical models for the nanoscale layered structures are also applicable to macroscopic layered structures [31]. In-plane tensile mechanical behaviors of monolayer graphene, interlayer shear behaviors as well as bending behaviors of GLNs, are introduced in Section 3.1, Section 3.2 and Section 3.3. Then, the conformations of large-scale graphene sheets (also denoted as graphene macromolecules) in solution, which may affect the microstructures of graphene assemblies, are also discussed in Section 4.1. Three types of graphene assemblies are introduced in Section 4.2, Section 4.3 and Section 4.4. Next, graphene/polymer nanocomposites with different microstructures are discussed in Section 5. Then, multi-scale strategies to optimize the mechanical properties of graphene-related materials are summarized in Section 6. Finally, we provide a summary and outlook for further works of GLMs in Section 7.

## 2. Theoretical Models of GLMs

### 2.1. Theoretical Models for Tension Behaviors of Monolayer Graphene

**Figure 2:**
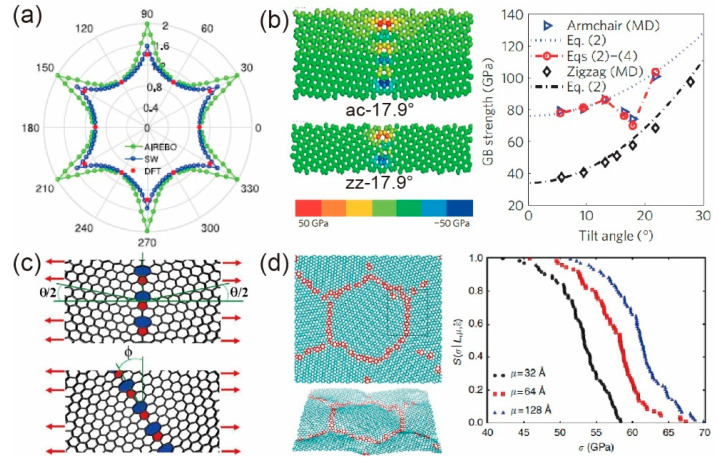
In-plane mechanical behaviors of monolayer graphene. (**a**) Anisotropic toughness of pristine graphene. Reprinted with permission from [66]. (**b**) Initial stress field induced by grain boundaries with different tilt angles along armchair (ac) and zigzag (zz) directions, and the strength-tilt angle relation. Adapted and reprinted with permission from [67]. (**c**) Schematic plot of two grains showing GB tilt angle (*θ*) and tensile loading angle (*φ*). Reprinted with permission from [68]. (**d**) Top and perspective views of graphene polycrystalline, and the survival probability predicted by the statistic model. Reprinted with permission from [69].

The classical mechanical model may be inappropriate to describe the mechanical properties of the atomic thick 2D materials, thus it is important to develop new theoretical models to accurately predict the structure–property relations of 2D materials to design the mechanical properties of 2D materials. The in-plane Young’s modulus of graphene in a small deformation regime exhibits isotropy credit to the sixfold symmetry atomic structure, but the overall stress–strain relation, strength, and toughness are direction-dependent under finite deformations. For example, the ultimate stress along the armchair direction and zigzag direction has a variation of 11 GPa in terms of Cauchy stress using density functional theory (DFT) simulation [57], while are 6~26 GPa in MD simulations using different force fields [58,59,60,61,62], as listed in detail in Table 1. Furthermore, several continuum models based on hyperelasticity have been proposed [63,64,65] to elucidate the nonlinear behaviors of pristine graphene with finite deformation under uniaxial loading, biaxial loading, or nanoindentation [63], where the elastic constants are determined by least-squares fitting to the ab initio calculations [63,64,65]. In particular, Hossain et al. [66] found that cracks always nucleate and propagate along the zigzag direction under uniaxial tension in different directions. Based on this observation, they proposed a simplified formula to characterize the chiral dependence of strength and toughness, which fits the MD results very well by considering the non-linear elastic deformation, as shown in Figure 2a for anisotropic toughness.

According to the second law of thermodynamics, defects are inevitably introduced into monolayer graphene in the fabricating process [47,48]. Qin et al. [48] reviewed the effects of several types of defects including point defect (vacancies, dislocations, S–W defects), line defect (grain boundaries (GBs)), pattern defect (chemical functionalization), and areal defect (hole and crack) as well as the underlying mechanisms to fracture behaviors. However, few theories have been developed to predict the overall mechanical properties of defective graphene, which is quite a big challenge. Among the limited theoretical works, Read and Shockley [70] predicted the energies and motions of GBs between two crystallites using the dislocation model, which works well for small-angle GBs. Based on the disclination model [71], Li [72] deduced an elastic field that is induced by a disclination dipole and serves for both small-angle and large-angle GBs. Then, considering the interactions between disclination dipoles, and following the rules that failure always initiated at the bond (shared by the hexagon-heptagon rings) with maximum residual stress, Wei et al. [67] theoretically gave the relation of strength to the tilt angle (as shown in Figure 2c) of GBs under tension perpendicular to the GBs, as shown in Figure 2b, which is well consistent with the MD simulations. With the consideration of loading direction (as shown in Figure 2c), Fox et al. [68] systematically studied the failure strength of GBs under tension in all possible directions varying from 0° to 90° using continuum theory and MD simulations. Besides, Shekhawat and Ritchie [69] developed a statistic model to predict the strength and toughness of polycrystalline graphene containing GBs and triple junctions with different sample sizes, grain sizes, and strain rates, as shown in Figure 2d. Then, they obtained the scaling laws for both the strength and toughness of polycrystalline graphene, and predicted that the toughness is independent to grain sizes over 25.6 nm, while the fracture strength is always grain-size and sample-size dependent. Moreover, Meng et al. [73] investigated the dislocation shielding effect on crack propagation and predicted the fracture toughness enhancement using theoretical analysis and MD simulations, as shown in Figure 6g.

### 2.2. Theoretical Models for Tensile Behaviors of Nacre-Like Structures

**Figure 3:**
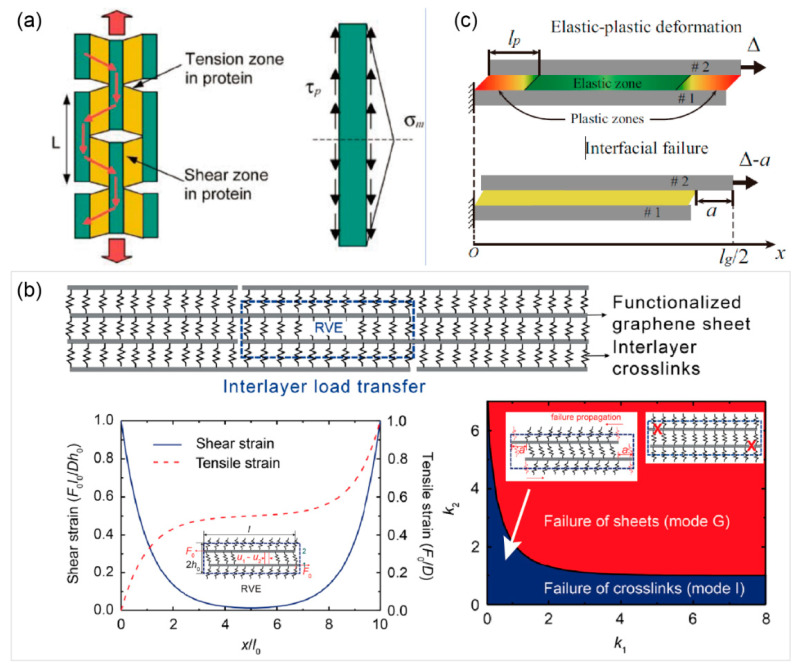
Tension–shear models for the nacre-like structure. (**a**) Tension–shear chain (TSC) model. The mineral tablets were assumed to be rigid, and the shear deformation of protein was the same everywhere. Reprinted with permission from [74]. (**b**) Deformable tension-shear (DTS) model. Two failure modes were distinguished, namely fracture of sheets (mode G) and failure of crosslinks (mode I). Adapted and reprinted with permission from [22]. (**c**) Nonlinear tension–shear model. The interlayer interactions are elastic perfectly plastic. Reprinted with permission from [23].

As aforementioned in Section 1, the in-plane tensile force is transferred to the adjacent platelet through interlayer shear stress for nacre-like GLNs. Therefore, it is necessary to develop a theoretical model to describe the tension-shear load transfer to further predict the mechanical properties of this kind of structure. As shown in Figure 3a, a tension-shear chain (TSC) model was first presented by Ji and Gao [74] to describe the mechanical behaviors of biological materials like nacre and bone, where the aligned mineral tablets are connected by soft and tough protein in a layer-by-layer manner. The TSC model reveals that tensile load in mineral tables is mainly transferred by the large shear zones of the protein matrix, so that the mineral tablets carry most of the tensile load while the protein matrix provides large shear deformation. As a result, the rigidity of minerals and toughness of protein are simultaneously maintained with an optimized aspect ratio of mineral, mineral content, and also the hierarchical structures. Then, Zhang et al. [75] developed a quasi-self-similar hierarchical model based on the TSC model, which gives the optimal levels for the hierarchical nacre-like materials (i.e., bone, mineralized tendon, and shell) and agrees well with natural observations.

By considering the tensile deformation of platelets, Liu et al. [21,22] developed a deformable tension-shear (DTS) model, as shown in Figure 3b to describe the tension behaviors of nacre-like GLNs, in which the extremely large aspect ratio of graphene sheet results in significant in-plane tension deformation of the platelet. Thus, a characteristic length scale is defined as $l_{0}=\sqrt{Dh_{0}/4G}$, where *D*, *h*_0_, and *G* are tensile rigidity of graphene sheet, interlayer distance, and interlayer shear modulus, respectively. When the length of graphene sheets *l* exceeds 3*l*_0_, the TSC model fails to describe the overall mechanical behaviors of nacre-like GLNs due to the tension of graphene sheets and nonuniform interlayer shear stress. Furthermore, two failure modes (i.e., the fracture of graphene sheets (mode G) and failure of interlayer crosslinks (mode I)), were distinguished. With the increase in *l*/*l*_0_, the tensile strength increases and converges quickly to a saturated value; when *l* exceeds 5*l*_0_, the stiffness also increases but converges at a larger length, and the toughness reaches the maximum value at about *l* = 3.3*l*_0_. Based on this understanding, the ultra-strong graphene fiber with giant graphene sheets was reported by Xu et al. [9]. 

The DTS model also revealed that the interlayer shear deformation is mainly located within the distance of *l*_0_ to the graphene edges, as shown in Figure 3b, which means that the most effective shear load transfer is at this region. Therefore, the interlayer crosslinks away from this region have limited contribution to the overall behaviors of GLNs. Then, to improve the contribution of interlayer crosslinks to the overall mechanical properties, noncovalent and self-healable crosslinks (i.e., hydrogen or ionic bond), which can reconstruct after breaking and prevent the quick propagation of interfacial break, can be introduced into the interlayer [26,27]. Accordingly, a nonlinear tension–shear model with consideration of the interlayer sliding was proposed by He et al. [23] to explore the strengthening and toughening mechanisms of noncovalent crosslinked GLNs, where the interface is assumed to be elastic perfectly plastic (as shown in Figure 3c), while the interface is elastic–brittle in the original DTS model.

### 2.3. Theoretical Models for Bending Behaviors of Laminar Structure

**Figure 4:**
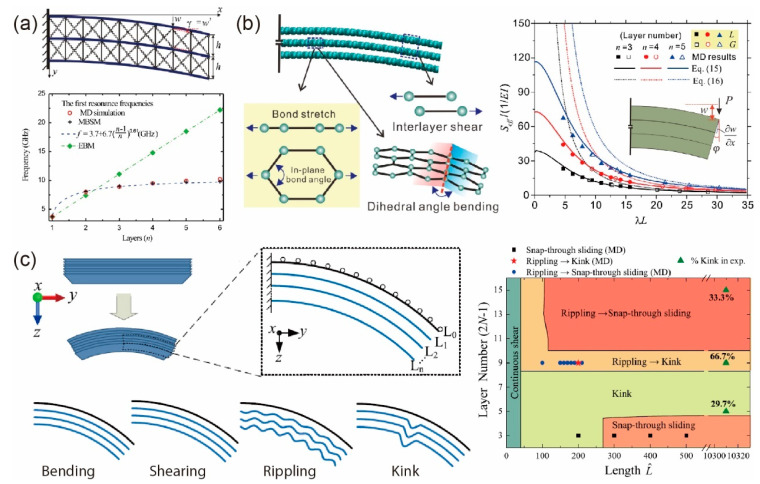
Bending models for laminar structures. (**a**) Multi-beam shear model, the in-plane extension of each sheet is neglected. Reprinted with permission from [78]. (**b**) Modified Timoshenko beam model (MTBM). Reprinted with permission from [31]. (**c**) Failure behaviors of GLNs under bending deformation, including interlayer shearing, rippling, and kink/delamination. Adapted and reprinted with permission from [32].

The bending behaviors of layered materials are quite different from traditional bulk materials due to the local curvature dependent bending energy of monolayer graphene and large interlayer shear deformation. Although the Timoshenko beam model can consider the shear deformation, it does not account for monolayer bending energy [76,77]. To solve this problem, Liu et al. [78] proposed a multi-beam shear model by considering monolayer bending energy and interlayer shear energy separately, as shown in Figure 4a, which could describe the bending behaviors of few-layer graphene with small interlayer shear modulus due to neglecting the in-plane extension in this model. Combining continuum theory and MD simulations, Shen and Wu [79] found that the bending rigidity was proportional to layer number for layer numbers exceeding five. Then, Liu et al. [80] proposed an improved beam model with in-plane tension by treating the layered materials as an architectured multi-beam. Based on a similar idea, the effects of interlayer plasticity and pre-delamination on bending rigidity of layered materials were also studied [29]. Recently, by introducing monolayer bending energy into the potential energy of the Timoshenko beam model, Qin et al. [31] developed a modified Timoshenko beam model (MTBM) that can correctly predict the bending stiffness of layered materials and structures without any fitting parameters, as shown in Figure 4b. They found that the bending behaviors of layered materials can be determined by a dimensionless parameter *λL*, where *L* is the length of the beam and $\lambda =\sqrt{kGA/D_{0}+kGA/\left(nD_{bend}\right)}$; *kGA* and *D*_0_ are the shear and bending rigidity of the beam cross-section, respectively; *D*_bend_ is the bending rigidity of monolayer; and *n* is the number of layer. They further performed MD simulations, finite element simulations, and experiments to validate MTBM. This model captures the intrinsic deformation modes of layered materials to provide an accurate tool to predict and optimize the mechanical properties of layered materials. Furthermore, the failure behaviors of layered materials under bending deformation have also been theoretically studied by Pan et al. [32]. Three failure modes including interlayer shearing, rippling, and kink delamination were demonstrated with length- and thickness-dependence, as shown in Figure 4c.

### 2.4. Theoretical Models for Out-of-Plane Deformations of GLMs

**Figure 5:**
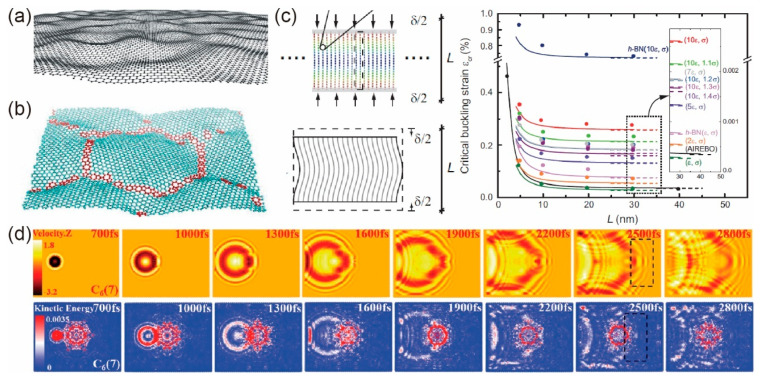
Out-of-plane deformation of GLMs. (**a**) Intrinsic rippling. Reprinted with permission from [14]. (**b**) Buckling induced by grain boundaries. Reprinted with permission from [69]. (**c**) Intrinsic buckling of GLNs induced by external load parallel to the sheet direction. Reprinted with permission from [33]. (**d**) Wrinkle propagation through flower-like GBs. Reprinted with permission from. [86].

Out-of-plane deformation of pristine graphene can be induced by thermal fluctuation [81,82]. Due to the atomic thickness, the bending rigidity of monolayer graphene is treated as an independent material parameter related to the bond angle and out-of-plane dihedral angle deformation [31], which is approximately one-tenth of that predicted by the classical beam theories (assuming the thickness as 0.335 nm [83]). Therefore, monolayer graphene is more likely to bend than stretch, which results in the spontaneous ripples of suspended graphene sheets under finite temperature that have been widely observed in experiments [84], as shown in Figure 5a, and further affects the in-plane and out-of-plane mechanical performances. With this scenario, different physical mechanisms have been proposed to explain the rippling. For example, Gao and Huang [85] developed a statistical mechanics model to explain the effects of thermal fluctuation on the elastic properties of monolayer graphene. They found that due to thermal fluctuation, the in-plane stress increases nonlinearly with applied strain even in the infinitesimal region, in contrast to the classical linear elastic theory.

Out-of-plane deformation of graphene can also be induced by defects (as shown in Figure 5b) and external load (as shown in Figure 5c) [33,34,87]. Based on the observation that the length of C–C bonds increases around 10% when attached by the OH group [88], Rebea et al. [89] indicated that a 20% concentration of OH adsorbates can generate ripples with the experimental observed wavelength and amplitude. On the other hand, in-plane defects within graphene sheets would introduce residual strain energy, which can be partly released by out-of-plane deformation. With this scenario, Elder et al. [90] proposed an extended phase field crystal model that was employed to investigate the out-of-plane deformation induced by strain, dislocation dipoles, and grain boundaries. Besides, this model can also capture the out-of-plane deformation of stacked bilayer materials. In terms of external load, Ren et al. [33] indicated the intrinsic buckling of layered materials without structure slenderness, as shown in Figure 5c. The critical buckling strain depends only on several elastic constants of the layered materials when the size of layered materials along the compression direction exceeds a certain length. The continuum model can describe the behaviors very well. In contrast, the critical buckling load of the monolayer and bilayer graphene sheet was found to be dependent on size, boundary, aspect ratio, and chirality [34]. Moreover, by considering of vdW interactions, post-buckling modes of monolayer graphene under compressive loadings were found to be qualitatively different from that of the planar Euler elastica [35].

Furthermore, the impacting induced out-of-plane wrinkle propagation in graphene sheets was also studied [86]. It is found that in graphene sheets with flower-like grain boundaries, the curvatures mismatch between the wrinkles, and the flower-like GBs can generate a significant defect shielding/offsetting effect for wrinkle propagation. Otherwise, if their curvatures are similar, dynamic wrinkles can pass the flower-like GBs without a significant shielding effect, as shown in Figure 5d.

## 3. Mechanical Behaviors of Graphene at Nanoscale

### 3.1. Mechanical Behaviors of Monolayer Graphene

The elastic stiffness *E*_2*D*_ and intrinsic strength of freestanding graphene were first measured by nanoindentation with an atomic force microscope (AFM) with values of 340 N/m and 130 GPa, respectively [83]. Then, Zhang et al. [91] found that the fracture toughness of graphene was about 15 J/m^2^, comparable to that of brittle materials [92,93]. Another definition of material toughness is the strain energy stored before failure, which for pristine graphene is 24.0 J/m^3^ along the armchair direction obtained from DFT simulations [66]. Here, we mainly focused on the effect of defects on the in-plane mechanical properties of monolayer graphene including modulus, strength, toughness, and fracture toughness as the graphene constituent in GLMs is defective.

**Figure 6:**
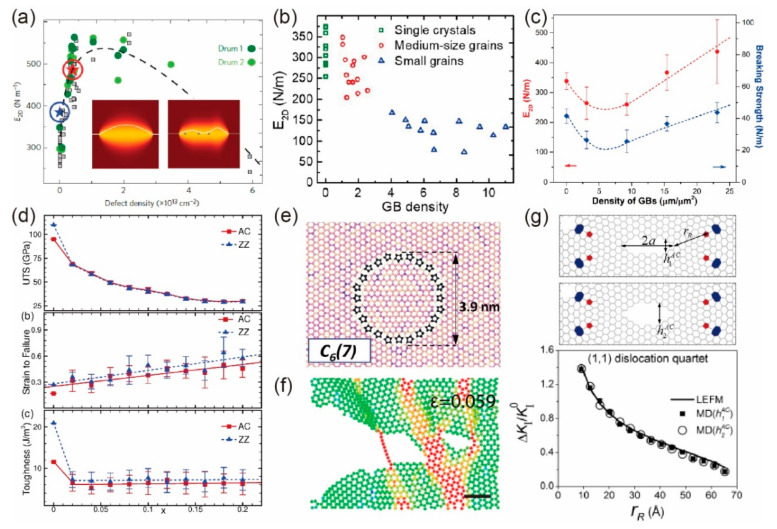
In-plane mechanical properties of defective graphene monolayer. (**a**) 2D elastic modulus of monolayer graphene as a function of vacancy density observed in the experiments. Adapted and reprinted with permission from [20]. (**b**) 2D elastic modulus as a function of GB density. Adapted and reprinted with permission from [98]. (**c**) 2D elastic modulus and strength as a function of GB density. Adapted and reprinted with permission from [100] (**d**) Strength, failure strain, and toughness of vacancy defected monolayer graphene as a function of vacancy density. Reprinted with permission from [97]. (**e**) Flower-like grain boundary. Reprinted with permission from [101]. (**f**) Atomic-scale crack bridging. Reprinted with permission from [102]. (**g**) Shielding effect of dislocation to crack propagation. Reprinted with permission from [73].

Most of the theoretical and simulated works have revealed that the vacancy defect decreases both the elastic stiffness *E*_2*D*_ and strength of graphene [94,95] because defects would induce residual stress in graphene [67], which is the atomic-scale origin of the decrease in modulus, strength, and toughness [96,97]. However, for *E*_2*D*_, López-Polín et al. [20] reported a counterintuitive fact that with the increase in defect density, *E*_2*D*_ first increases, and then decreases, leading to an elastic stiffness up to 550 N/m at vacancy density around 0.2%, as shown in Figure 6a. They attributed the initial increase in *E*_2*D*_ to the suppression of out-of-plane fluctuations by defects, which was not considered in former theories and simulations. Recently, Ruoff and co-authors have shown that *E*_2*D*_ of polycrystalline monolayer graphene decreases to an average value of ~120 N/m with the grain size of 1–2 μm [98], as shown in Figure 6b, which was also attributed to the out-of-plane deformation induced by GBs [99]. By continuing to decrease the grain size (increase GB density), Xu et al. [100] achieved an *E*_2*D*_ of 436 N/m with an average grain size of 20 nm, even higher than that of pristine graphene, as shown in Figure 6c.

In terms of strength, defective graphene with a vacancy density of 20% still possesses a fracture strength higher than that of most typical engineering materials [103,104], as shown in Figure 6d. The strength of polycrystalline graphene with tilt GBs is found to be sample size-, grain size-, and strain rate-dependent [67,69,100,105]. A conflict should be pointed out that, in statistical theory [69], the strength of polycrystalline graphene decreases with the decrease in grain size. However, in the experiment [100], the strength (also modulus) first decreases and then increases to a value comparable to that of pristine graphene, as shown in Figure 6c, indicating novel stiffening and strengthening mechanisms that have not been considered in previous theories. Furthermore, a recent study has shown that the strength of flower-like GBs (as shown in Figure 6e) is weakened by decreasing the curvature of the grain boundary [101].

The fracture behavior of monolayer graphene sheets was found to show a brittle-to-ductile transition with vacancy densities of 8–12%, and the toughness was found to dramatically decrease when introducing vacancy defects, but almost kept constant as the vacancy densities ranged from 2 to 20% [97]. On the other hand, the fracture toughness of pre-cracked graphene was widely found to be strongly affected by defects [73,102,106,107,108,109]. For example, the fracture toughness can be significantly enhanced by atomic-scale crack bridging, as shown in Figure 6f, where the single-atom chains continue to bridge fracture surfaces as the crack propagates [102]. Besides, the dislocation shielding effect on nano-cracks [73] was also revealed to improve the fracture toughness, as shown in Figure 6g.

### 3.2. Bending Behaviors of Multilayer Graphene

**Figure 7:**
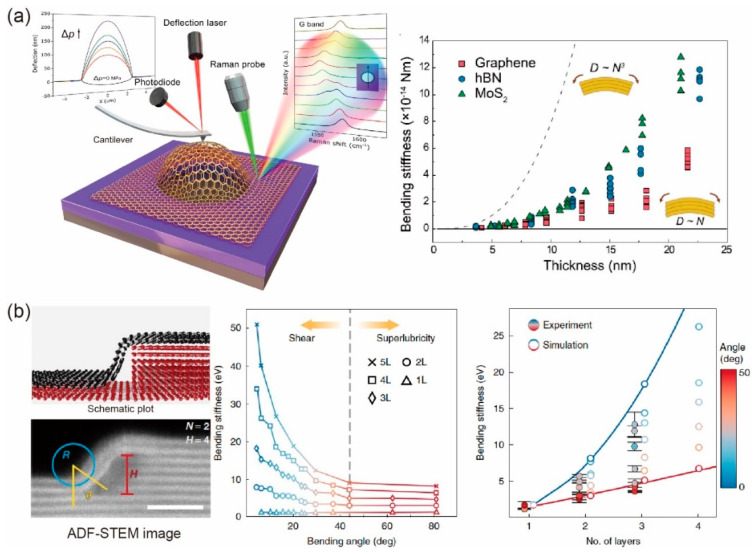
Bending behaviors of multilayer graphene. (**a**) Direct measurements of bending rigidity of multilayer graphene, molybdenum disulfide (MoS2), and hexagonal boron nitride (hBN) based on pressurized bubbles. Reprinted with permission from [111,112]. (**b**) Measurements and results of bending rigidity of multilayer graphene as a function of bending angle. Reprinted with permission from [30].

The bending rigidity of multilayer graphene has been found to be significantly enhanced by the weak interlayer shearing via nanoindentation and pressurized bubble experiments. It should be noted that the interlayer shear stiffness is three orders of magnitude smaller than that of intralayer stiffness, thus the relation of bending rigidity *D* to layer number *N* is predicted in the range of free interlayer sliding *D*~*N* to perfectly bonded *D*~*N*^3^. For example, Chen et al. [28] investigated the bending stiffness of few-layer graphene with self-folding conformation using atomic force microscopy (AFM) and nonlinear mechanical modeling. They found that the relation of the bending stiffness and layer number *N* (*N* = 2~6) followed *D*~*N*^2^, indicating that the weak interlayer shear interaction had a substantial stiffening effect for multilayer graphene. In contrast, Shen and Wu [79] found that *D* was proportional to *N* for *N* > 5, which can be extended to other kinds of 2D layered materials [110]. Wang et al. [111,112] used pressurized bubble experiments to directly measure the bending rigidity of layered materials, and similar laws of bending rigidity were found, as expected from previous works, as shown in Figure 7a. Furthermore, Han et al. [30] found the bend softening phenomenon of few-layer graphene, where the bending stiffness significantly decreases as the bending angle increases, as shown in Figure 7b. They attributed the softening mechanism to interlayer shear, slip, and the onset of superlubricity between adjacent layers.

### 3.3. In-Plane Mechanical Behaviors of Multilayer Graphene

In-plane mechanical properties of multilayer graphene, especially that with nacre-like nanostructures, are also strongly dependent on the interlayer properties. As the graphene sheet is discontinuous in nacre-like nanostructures, the in-plane load is transferred by interlayer shear stress to adjacent graphene sheets. Therefore, the interlayer shear properties play important roles in the overall mechanical properties. Intrinsic vdW interaction between graphene layers is too weak to carry enough in-plane load, thus interlayer crosslinking and nano-structural modification are introduced to regulate the interlayer shear modulus and strength.

**Figure 8:**
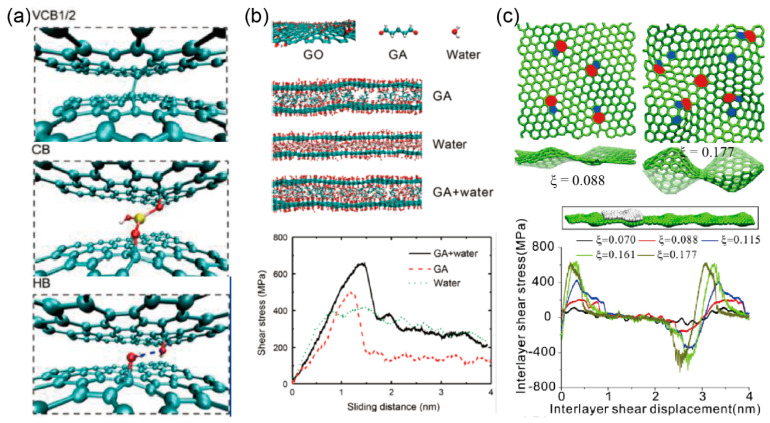
(**a**) Interlayer crosslinks including covalent (VCB1/2) and non-covalent (CB and HB) bonds. Reprinted with permission from [119]. Interlayer crosslinks (**b**) and geometrical locking (**c**) can improve the in-plane shear properties of multilayer graphene. Reprinted with permission from [22,120].

In general, modulus, strength, and toughness of multilayer graphene can be simultaneously improved by enhancing interlayer properties using typical interlayer bonds, as shown in Figure 8a for instance. To increase modulus and strength, covalent bonds should be introduced because they are stiff and strong. For example, if the two graphene layers are arranged in the A–B stacking phase, a covalent crosslink between can be built through the monovacancy in each layer [113,114], while in the A–A stacking phase, strong interlayer covalent bonds can be built with an interlayer distance of 0.156 nm [24]. Otherwise, in order to increase the toughness, non-covalent bonds including hydrogen [26,115], ionic [116], coordinate [117], and π–π bond [118] can be introduced due to their reconfigurability, which can prevent interlayer crack propagation. Moreover, the fracture toughness of functionalized graphene multilayers was experimentally measured to be more than two times higher than the graphene monolayer, which was attributed to the random distribution of functionalized carbon atoms that restrict the growth of a preexisting crack [107].

Since pristine graphene is insoluble and intractable, it is difficult to be shaped in desired structures, or be connected through interlayer interactions. Therefore, chemical modifications are used to generate graphene oxide (GO) and reduced graphene oxide (rGO) [121], which can produce a big number of oxygen-rich functional groups and generate rich interlayer interactions covalently or non-covalently, and further improve the overall mechanical properties, as shown in Figure 8b. Additionally, Qin et al. [120] demonstrated a geometrical locking effect of wrinkled graphene generated by topological defects to improve the interlayer shear properties. They found that the interlayer shear modulus was significantly increased with the increase in the aspect ratios of the wrinkles, as shown in Figure 8c.

## 4. Structure–Property Relations of Graphene Assemblies

Macroscopic graphene assemblies possess hierarchical microstructures that extend from the nanoscale to meter/centimeter scale. The monolayer graphene sheets first stack in a layer-by-layer manner to form the building blocks of GLNs, then GLNs can be assembled to the hierarchically layered structures or nanoporous structures. According to different macroscopic morphologies, these graphene assemblies can be distinguished into fiber (1D), film/paper (2D), and porous (3D) assemblies, respectively. Since the overall properties of materials are decided by their microstructures, the conformations of monolayer and multilayer graphene within graphene-based materials also play fundamental roles in the overall mechanical properties of macroscopic graphene assemblies. Thus, in this section, we first briefly introduce the conformations of GO sheets in solutions, then focus on the microstructures and mechanical properties of the aforementioned three types of graphene assemblies as well as the related fabricating and optimizing methods.

### 4.1. Conformations of GO Macromolecules

Monolayer GO sheets were experimentally processed into various conformations (i.e., the flat, crumpled, folded, and compact structures). For example, crumped GO nanosheets can be prepared by rapidly drying the aerosol, possessing a fractal dimension of 2.54 ± 0.04 [122]. Crumpled and compact conformations can be observed in dispersions by tuning the qualities of solvents [123,124,125] or introducing long-range interactions [126]. Folded conformation can be obtained by introducing ions (i.e., Ca^2+^) as short-range interaction in GO sheets with slight out-of-plane corrugation [126]. More recently, Li et al. [127] constructed a general description of thermodynamic and rheological behaviors of 2D macromolecules dispersed in solutions based on experimental and dissipative particle dynamics simulations, which provides a fundamental framework that can be used in the understanding and control of their assembly and processing in solution toward applications.

**Figure 9:**
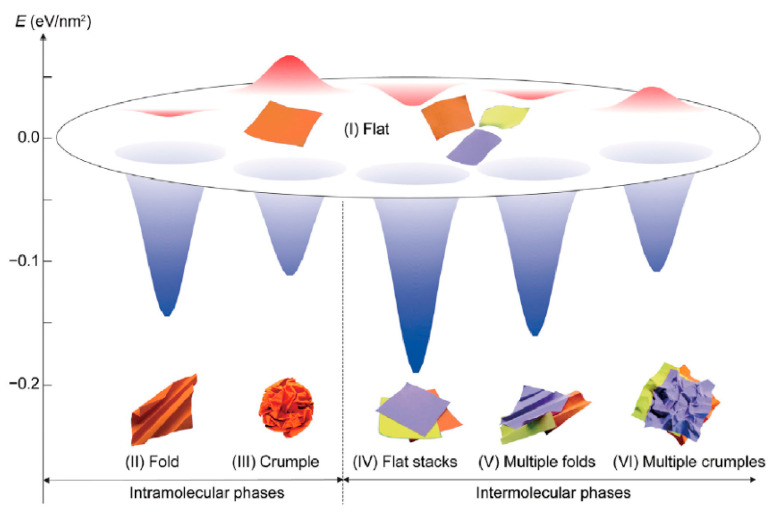
Conformation phase map of GO in solution accompanying the potential energy landscape. Reprinted with permission from [126].

More conformations have been observed in GO aqueous dispersions with high GO concentration, as shown in Figure 9. For example, GO sheets stay flat in good solvents (i.e., pure *N*, *N*-dimethylformamide (DMF)) and distribute uniformly and isotropically at low GO concentration (<0.02 mg/mL) [125]. However, the increase in GO concentration leads to a regular alignment of GO sheets in the nematic phase. Furthermore, a coexisting phase consisting of the isotropic distributed phase and nematic phase were also observed [125] at intermediate GO concentration. In contrast, inter-molecular attractions can be enhanced by introducing a poor solvent like ethylacetate (EA). For example, in extremely low GO concentration, GO sheets transit into the folded phase by introducing additional ions as short-range attractions, while turn into a crumpled phase with long-range attractions. Increasing GO concentration in poor solvent makes flat GO sheets stack together and form a flat-stack phase [126,128], while the folded GO sheets also stack and form into a multi-folded phase. Moreover, with long-range attractions, flat GO sheets first stack together and then shrink into a multiple-crumpled phase [126].

### 4.2. 1D Graphene Fiber Structures

**Figure 10:**
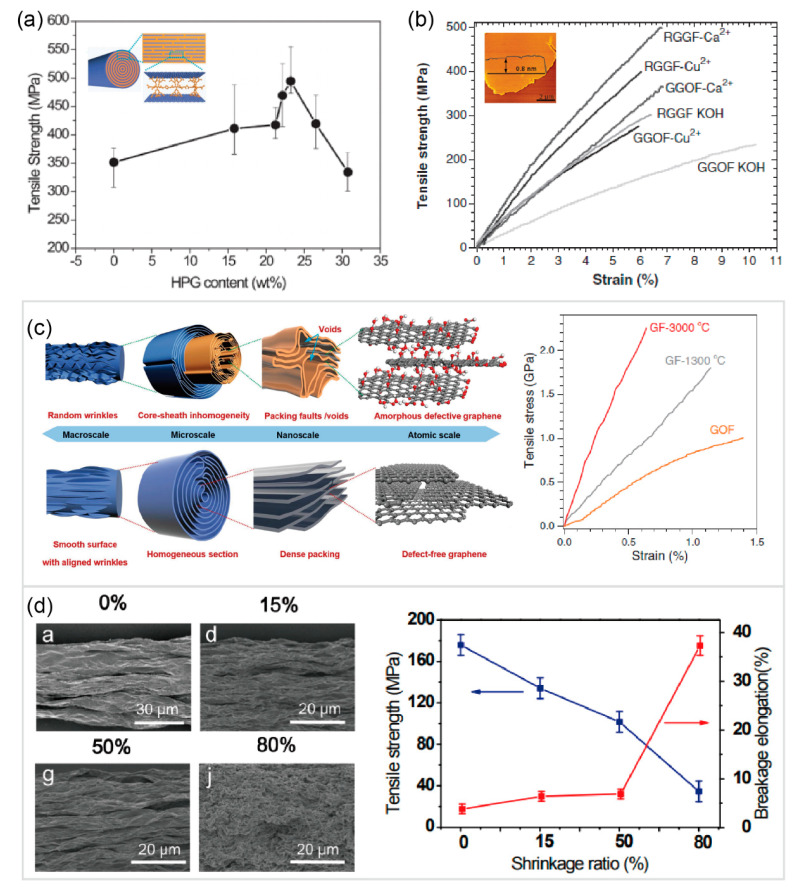
Graphene fibers (GFs). (**a**) Improved tensile strength for GFs with introduced polymers among adjacent sheets. Reprinted with permission from [132]. (**b**) Tensile stress curves for GFs with giant graphene sheets and different crosslinks. Reprinted with permission from [9]. (**c**) Schematic plot of full-scale synergetic defect engineering to manage possible defects of GFs from the atomic scale to macroscale and tensile stress curves of resultant GFs. Reprinted with permission from [133]. (**d**) Surface morphologies of shrunk GFs fabricated by dry-spinning method and corresponding strength and break elongation. Reprinted with permission from [129].

Graphene/GO fibers have attracted growing interest in the past decade [53]. Xu and Gao [12] fabricated fiber-like graphene assemblies using a wet-spinning process with GO chiral liquid crystals (LCs), which paves new ways for high-performance graphene-based materials. Then, diverse methods (i.e., wet-spinning [12], dry-spinning [129], confined hydrothermal strategy [130], and twisting films [131]) have emerged to prepare graphene/GO fibers. Graphene/GO fibers obtained from the aforementioned methods usually possess high porosity and non-uniform core-shell structure, hindering their excellent mechanical properties. Therefore, the crosslinking or wrinkle modulation methods were developed to further improve the mechanical properties of graphene/GO fibers. Hu et al. [132] proposed a self-templating methodology to introduce the polymer into the interspace of adjacent GO sheets during the wet-spinning process. The resultant GO–polymer fiber composites had a tensile strength 60% higher than that of neat GO fibers, as shown in Figure 10a. Xu et al. [9] found that the introduction of divalent ions enhanced the tensile modulus and strength to two times those without crosslinks. They also claimed that the giant size of graphene sheets considerably improved the mechanical performance, as shown in Figure 10b. On the other hand, more efforts have been spent on controlling the morphologies of graphene/GO sheets. For example, as schematically shown in Figure 10c, Xu et al. [133] presented a full-scale defect engineering method to minimize the possible defects including voids, inhomogeneity, random orientation, and wrinkles of graphene sheets, which created a new record of modulus for GFs up to 400 GPa. Xin et al. [134] obtained a high-performance graphene belt by controlling the alignment and orientation of graphene sheets in a microfluidic tube. Moreover, they also developed graphene fibers with more regular orientations by introducing small-sized graphene sheets to connect the large-sized ones [135]. By mimicking the microstructures of nacre, Cheng and co-authors prepared super tough yet strong graphene fibers via sequential interfacial interactions [136,137,138]. More recently, Li et al. [37] presented a plasticization spinning method that flattens random graphene wrinkles and regulates sheets with high order and stacking density, thereby forming large crystallite domains and demonstrating a new record of strength for GFs up to 3.4 GPa.

Here, it should be noted that most of the graphene fibers fabricated by other methods have lower modulus and strength than that of the wet-spinning method, but have higher breakage strain. For example, Tian et al. [129] found a significant shrinkage in both axial and radial directions of graphene fibers using the dry-spinning method, as shown in Figure 10d. The shrinkage in the axial direction, followed by the transition of partial orientation into wrinkles, produced a large failure strain up to 35%. Furthermore, Fang et al. [131] twisted a GO belt to form a graphene fiber with helical microstructures that could be stretched to a strain up to 29% until failure. Moreover, GO porous fibers have also been developed by combining the spinning and ice-template strategies [41]. More details for the mechanical properties of GFs can be found in Table 2.

### 4.3. 2D Graphene Thin Films

The graphene thin films were first fabricated as GO paper using a flow-directed assembly of individual GO sheets by Ruoff and co-authors in 2007 [36]. Subsequently, in 2008, Li et al. [11,146] first prepared pure graphene paper with chemically converted graphene (CCG) sheets from GO sheets. Primary fabrication approaches to obtain paper-like graphene assemblies include infiltration-aided assembly, solution casting, and spray coating, etc., which usually tend to bring out lamellar ordered GO sheets with random wrinkles [36] due to the disturbance, shrinking, and capillary contraction of GO sheets. Therefore, two directions to regulate the mechanical properties of graphene papers were taken, one is stiffening and strengthening, while the other is improving flexibility. However, the two pathways are usually contradictory.

**Figure 11:**
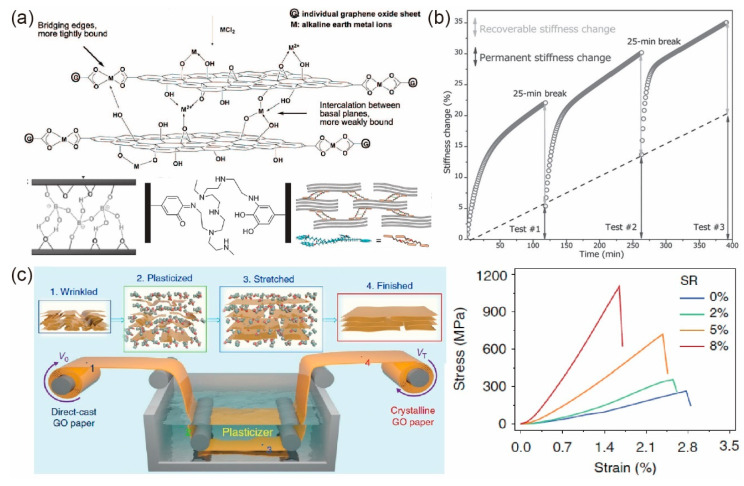
Stiffening and strengthening strategies for graphene papers (GPs). (**a**) Schematic plots of crosslinks introduced to adjacent sheets including (top) divalent bonds, (left to right in the bottom row) borate, polymers, and π–π crosslinking. Reprinted with permission from [142,147,148,149]. (**b**) Self-stiffening approach by cyclic stretching with small strain amplitude. Reprinted with permission from [151]. (**c**) Plasticizer-assistant stretching method to straighten the wrinkles of direct-cast GO papers, and the resultant stress–strain curves. Reprinted with permission from [143].

Stiffening and strengthening strategies involve the crosslinking of adjacent graphene/GO sheets [140,142,147,148,149,150] and wrinkle straightening [143,151]. Different kinds of crosslinkers including divalent ions [147], borate [148], polymers [149,150], π–π crosslinking [140,142], etc., as schematically shown in Figure 11a, were proven to efficiently enhance the stiffness and strength of GO paper [21,22,23]. On the other hand, Dai et al. [151] observed a significant enhancement in modulus of the GPs up to 84% by applying cyclic dynamic loading at a low strain amplitude of 0.1% (see Figure 11b), which was attributed to the straightening and reorientation of GO sheets in dynamic loading. Based on a similar idea, Wan et al. [145] proposed a freeze stretching strategy where static bi-axial load was applied to the sample, and covalent and π–π bonds were introduced to the inter-platelet space, which produced a strength up to 1.55 GPa. Furthermore, by introducing solvent plasticizers, Li et al. [143] developed a plasticizer-assistant stretching method to straighten the wrinkles of direct-casted GO papers. They found that the tensile modulus and strength of the modified GO papers were significantly increased up to 693% and 370% compared to those of un-straightened GO papers, respectively, as shown in Figure 11c.

**Figure 12:**
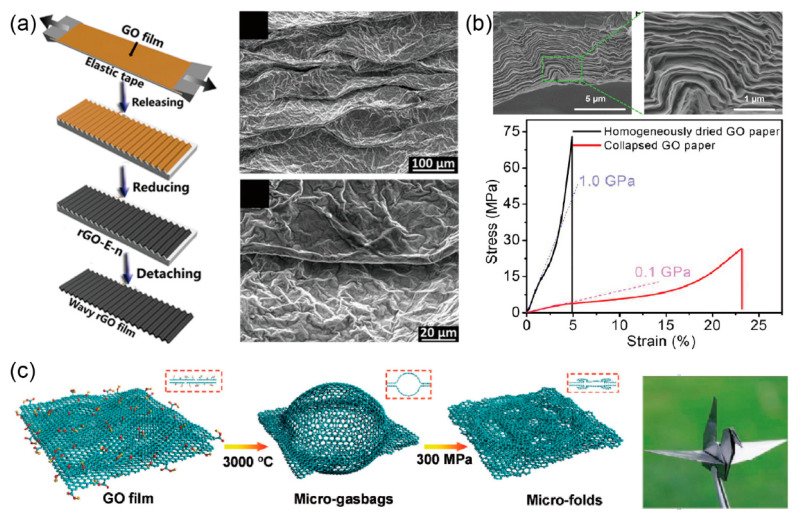
Improving the flexibility of graphene papers (GPs). (**a**) Surface wrinkling method and top view of the resultant wrinkle morphology. Reprinted with permission from [153]. (**b**) Rubber-like GPs with hierarchical crumples obtained by sheet collapsing approach. Reprinted with permission from [125]. (**c**) Thermal annealing approach and resultant highly flexible GPs with micro folds. Reprinted with permission from [141].

On the other hand, by intentionally introducing micro wrinkles and folds, the flexibility of GPs was significantly improved [125,141,152,153,154,155,156]. For example, a promising method used to introduce surface wrinkling patterns is to release the pre-strained polymer substrate coated with large-area graphene film [152,153,154,155], as shown in Figure 12a. In order to form wrinkle or fold patterns, three main steps should be taken including stretching the polymer substrate first, then coating few-layered graphene [154] or GO dispersion [152,153] on the substrate, and finally, releasing the stretched substrate in a controlled way. Furthermore, additional reducing and transfer methods [157] should also be employed for further usage of the graphene films with designed wrinkles. Beyond surface wrinkling, one can also introduce wrinkles and folds as the intrinsic microstructures of freestanding paper-like graphene assemblies. For example, Liu et al. [156] obtained flexible graphene papers with micro folds by mechanically compressing graphene aerogels. Xiao et al. [125] soaked jelly-like blade-casted GO/DMF film into a poor solvent (EA) pool to promote the collapse of GO sheets, followed by hang-drying under self-weight. This sheet-collapsing process in poor solvent resulted in a rubber-like GO paper with hierarchical crumples whose breakage elongation increased up to 23%, as shown in Figure 12b. Furthermore, Peng et al. [141] proposed a thermal annealing approach to introduce micro folds into GO papers. During the annealing process, gasbags are formed with sizes from several to tens of micrometers. Under high compression, micro folds are then introduced to the resultant graphene paper with high flexibility, as shown in Figure 12c. More details on the mechanical properties of GPs can be found in Table 2. In addition, nano wrinkles were found to play important roles in mass transport across graphene-based films [158,159].

### 4.4. 3D Graphene Porous Structures

**Figure 13:**
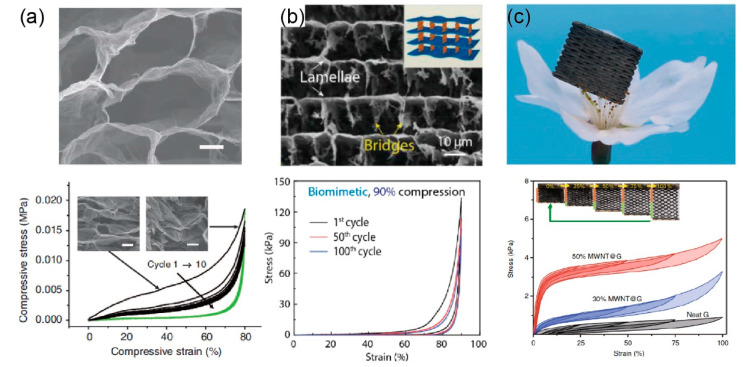
Graphene aerogels (GAs). (**a**) Regular microstructures of GAs resultant from template directing method. Reprinted with permission from [163]. (**b**) Bio-mimicking lamellar GAs with exceptional strength and mechanical resilience. Reprinted with permission from [165]. (**c**) 3D printed stretchable GAs. Reprinted with permission from [164].

Graphene porous structures, which are called graphene aerogels (GAs) or graphene foams, were fabricated [38,160,161,162]. The lowest density of GAs ever reported up until now is 0.16 mg/cm^3^ [10], which is even lighter than air. To eventually obtain GAs, their precursors (like graphene hydrogel), the composites of graphene networks and solvent should first be prepared, followed by drying of the precursors to remove the solvent. In general, methods to obtain the precursors include hydrothermal reduction, chemical reduction, crosslinking, and template directing. Here, methods of reduction and crosslinking can promote the self-assembly of graphene sheets, and highlight the random distribution of microporous structures in graphene networks, while the method of template directing can prevent random interconnection and produce more regular microstructures [163], as shown in Figure 13a. It should be noted that the bulk GAs fabricated from the aforementioned methods are highly compressible but less stretchable. More recently, a 3D printing process has been employed to fabricate the GA lattice, whose mechanical properties depend not only on the microporous structures, but also on the macro lattice structures, thus providing mechanical designability to graphene 3D assemblies (i.e., the stretchable GAs [164]), as shown in Figure 13c.

In general, the stress–strain curves for GAs under compression can be divided into three regimes including the nearly linear elastic regime, stress plateau regime, and stress densification regime, corresponding to bending of the cell walls, buckling of the cell walls, and densification of the cells, respectively. Unlike the stretching of graphene sheets in the paper- or fiber-like assemblies, the bending of cell walls results in an extremely low initial modulus smaller than 1.0 MPa. Furthermore, hysteresis can also be found during cyclic loading of GAs, which is attributed to the discontinuous buckling and breakage of connections among the graphene sheets. Here, the breakage of the internal structure and the irreversible buckling in GAs could also induce the degradation of elasticity, modulus, and strength. To solve these problems, Qiu et al. [166] proposed a thermal annealing strategy to improve the inter-sheet interactions, which produces the complete recovery of GAs from 98% compression. Furthermore, by mimicking biomaterials, Yang et al. [165] developed 3D interconnected lamellar GAs with exceptional strength and mechanical resilience, as shown in Figure 13b.

Due to the hierarchical porous structures, GAs are probably more promising for multi-functional applications such as elastic and flexible conductors, environmental protection, energy storage, sensors, supercapacitors, and catalyst beds, etc. [167,168,169,170,171,172], rather than mechanical load-bearing.

## 5. Structure–Property Relations of Graphene/Polymer Nanocomposites

Composites have attracted lasting interest during the past decades, which are consisted of two or more constituent materials with advanced mechanical properties compared to those of the constituent materials. The mechanical properties of composites are related to their matrix, fillers, and interfaces. Due to the excellent Young’s modulus, strength, thermal, and electrical conductivities, graphene is an ideal nanofiller in high-performance nanocomposites with mechanical properties superior to the polymer matrix. However, among the ever-published papers, the mechanical properties of nanocomposites with a high content of graphene are usually compared with pure graphene assemblies rather than polymers. With this scenario, we first focused on the graphene/polymer nanocomposites with a relatively low content of graphene, whose microstructures and mechanical behaviors compared to the pure polymers are discussed in Section 5.1, Section 5.2, Section 5.3. For paper-like nanocomposites with a high content of graphene, their mechanical properties compared to pure graphene assemblies are presented in Section 5.4. The up-to-date works on the mechanical properties of graphene/polymer nanocomposites compared to the pure matrix are summarized in Table 3.

### 5.1. Graphene Dispersed in Polymer Matrix

**Figure 14:**
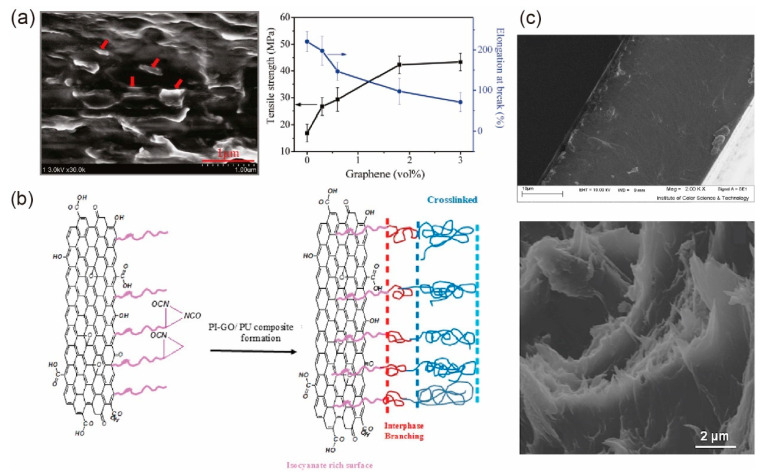
Graphene/polymer nanocomposites with graphene sheets dispersed in polymers. (**a**) Graphene sheets dispersed near randomly in polymers, enhanced strength and decreased break elongation. Reprinted with permission from [180]. (**b**) Introducing interfacial crosslinks (covalent bonds here) between graphene sheets and polymers brings out overall enhanced mechanical properties. Reprinted with permission from [43]. (**c**) Fractured surface of nanocomposites with covalent (**top**) and non-covalent (**bottom**) functionalized graphene sheets. Reprinted with permission from [43,183].

Composites with nanofillers dispersed in polymer matrix have been widely studied in the past decades. GO is a promising precursor for their rich functional groups, which can provide efficient interactions between GO sheets and polymers. Therefore, GO/polymer composites were first prepared, where GO is further reduced into rGO, and rGO/polymer composites are obtained in the end. In general, these kinds of composites are superior in Young’s modulus and strength compared to the pure polymers. For example, the tensile strength and Young’s modulus of rGO/PVA composites with graphene content of 1.8 vol% fabricated by Zhao et al. [180] are 1.5 and 10 times larger than that of pure PVA, as shown in Figure 14a. The comparison of Young’s modulus between experiments and theories derived from the Halpin–Tsai model [181] indicates that most graphene sheets are randomly distributed in nanocomposites. Furthermore, the modified Halpin–Tsai model [180,182] also revealed that the nanocomposites with aligned nanofillers exhibited a higher modulus than those with randomly distributed nanofillers. Therefore, methods [55] such as the infiltration and liquid crystal method have been proposed to fabricate aligned graphene/polymer nanocomposites [13,55].

Although the strength of composites with dispersed fillers is enhanced due to the shielding effect of nanofillers to crack propagation perpendicular to the fillers [184], the dispersions of graphene in polymer easily generate fracture at massive interfaces due to the weak vdW interaction. As a result, compared to pure polymers, nanocomposites with dispersed nanofillers usually show a decline in breakage elongations and toughness. To overcome this problem, non-covalent [173,175,183,185] and covalent [186,187,188] functionalization of GO sheets with polymer chains were proposed to enhance the interfacial interactions between the two components to further improve the mechanical properties of composites [42]. Here, covalent functionalization of GO usually provides stronger covalent bonds between the functionalized GO sheets and polymers, thus was expected to provide higher load transferring capacity than non-covalent bonds. For example, by attaching the polyisocyanate (PI) polymer through covalent bonds on GO sheets first, and then incorporating the modified GO sheets into PU matrix through covalent bonds between PI and PU, Ramezanzadeh et al. [43] found that the resultant PU/PI-GO composites (as shown in Figure 14b) had a pronounced enhancement in overall mechanical properties including Young’s modulus, tensile strength, break elongation, and toughness compared to neat PU and PU/GO composites. Here, it should be noted that a smooth fractured surface in PU/PI-GO composites (the top image in Figure 14c) observed by SEM microscopy [43] indicates that the overall fracture happens in the polymer matrix rather than the interface. In contrast, step morphologies were observed in the fracture surface of nanocomposites with non-covalent functionalized graphene sheets [183], indicating sliding between the GO sheets and polymer matrix (the bottom image in Figure 14c).

The weak vdW interaction among GO sheets plays an important role when the GO content rises to a relatively high value, leading to aggregation of the graphene sheets, which limits the mechanical enhancement of the nanocomposites. The aggregation can be partially solved by employing interfacial modification methods [176,189]. Moreover, it can also be solved via another method, infiltrating polymers into a pre-fabricated graphene scaffold, which will be discussed in Section 5.2.

### 5.2. Polymer Infiltrated into Graphene Scaffold

**Figure 15:**
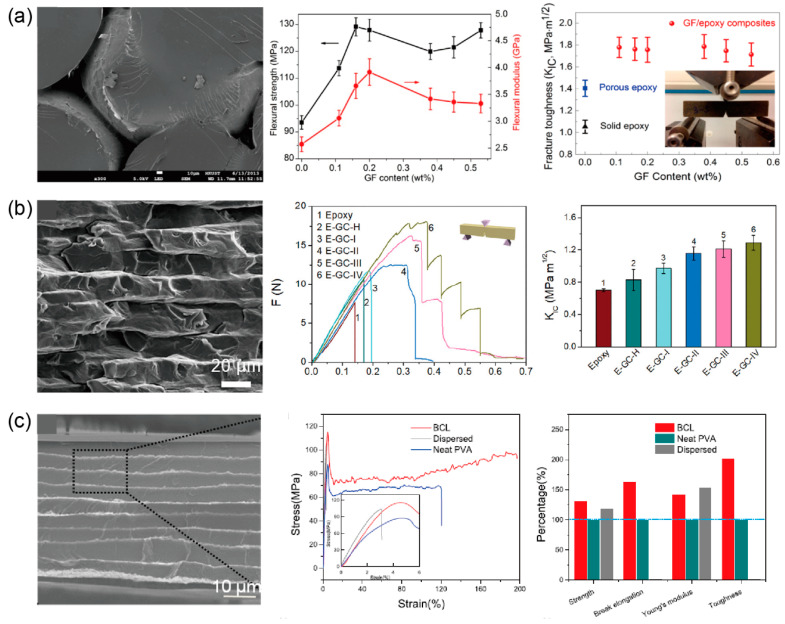
Graphene/polymer nanocomposites with polymer infiltrated into graphene scaffold and bi-continuous microstructure. (**a**) RGO/epoxy composites, in which the graphene scaffold was grown on a Ni foam template, showing enhanced flexural modulus, strength, and fracture toughness compared to pure epoxy. Reprinted with permission from [190]. (**b**) RGO/epoxy composites with well-aligned rGO-CMC (carboxymethyl cellulose sodium) scaffold by ice templating, showing enhanced fracture strength and toughness. Reprinted with permission from [45]. (**c**) Bi-continuous GO/PVA composites, showing overall enhancement of mechanical properties including Young’s modulus, strength, break elongation, and toughness compared to pure PVA. Reprinted with permission from [46].

This kind of nanocomposites can be achieved by forming a 3D graphene scaffold on the template and then infiltrating polymers into the scaffold. With this pre-scaffold-infiltration process, Ji et al. [190] prepared rGO/epoxy composites with the graphene scaffold grown on a Ni foam template. The flexural modulus and strength of the resultant rGO/epoxy composites were 53% and 38% larger than that of solid neat epoxy at 0.2 wt% of graphene content, and the fracture toughness was remarkably increased by 70% at 0.1 wt% of graphene content, as shown in Figure 15a. The toughening mechanism can be attributed to the combination of crack tip blunting, interfacial debonding, slipping, and separating between adjacent graphene layers, which provide enhanced energy dissipation in composites. Furthermore, as shown in Figure 15b, Cheng and collaborators [44,45] fabricated an inverse nacre-like layered epoxy nanocomposite by infiltrating the liquid epoxy precursor into the rGO-CMC scaffold. They found that the fracture toughness *K*_IC_ with pre-crack perpendicular to the layered structure could be enhanced up to 3.61 times to that of pure epoxy, while the strength exhibited anisotropy due to the anisotropy of the scaffold. Thanks to the continuous structure of graphene networks, composites made by this pre-network strategy also possess superior electric conductivity among graphene-based composites.

### 5.3. Graphene/Polymer Bi-Continuous Lamina Composites

As shown in Figure 15c, Liu et al. [46] proposed a continuous fracture toughening mechanism with bi-continuous laminar GO/PVA composites, which were fabricated using the blade-coating process with the GO layer and PVA layer aligned one by one. Due to the bi-continuous structure, both GO and PVA are subjected to pure tensile stress before the fracture of GO layers under stretching along the lamina direction. As a result, Young’s modulus of the composites can simply be determined by the superposition of the volume fraction of the two components. During stretching, massive cracks in GO layers occurred along the perpendicular direction, which led to the formation of lots of graphene fragments. After that, due to shear-lag effects between the still continuous PVA layer and discontinuous GO fragments, the sustaining fracture made the fragments further disintegrate into smaller sub-fragments. Once the size of GO sub-fragments is small enough that the interface sliding force cannot exceed the tensile strength of GO fragments, the sub-fragments stop breaking again, then the applied force increases monotonously, showing a strain hardening behavior until the fracture of PVA. With 0.3 wt% GO content, the mechanical properties of GO/PVA composites were significantly improved by 42.3%, 32.2%, 64.2%, and 101.4% in the Young’s modulus, tensile strength, break elongation, and toughness, respectively, compared to pure PVA. As a result, this bi-continuous structure, combined with the corresponding continuous fracture mechanism could provide an alternative methodology to simultaneously strengthen and toughen the nanocomposites.

### 5.4. Nacre-Like Graphene Nanocomposites

**Figure 16:**
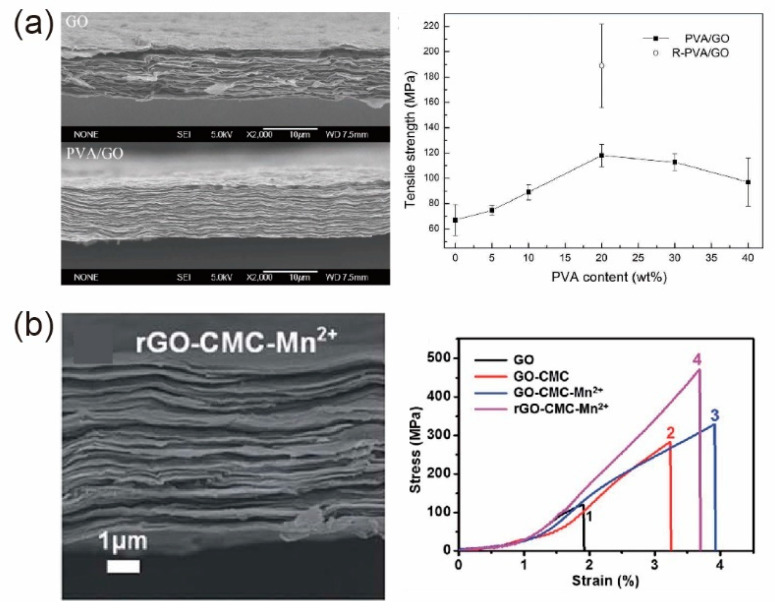
Nacre-like graphene/polymer nanocomposites. (**a**) PVA/GO composite films with enhanced tensile strength and fracture strain. Adapted and reprinted with permission from [191]. (**b**) GO (rGO)/CMC composites with improved mechanical properties. Adapted and reprinted with permission from [179].

By mimicking the natural nacre, nanocomposites with high graphene content (i.e., more than 50 wt%) were fabricated as nacre-like composites [177,179,191]. In contrast to those of low graphene content, graphene sheets in nacre-like composites serve as matric phases, which is similar in function to the “bricks” in natural nacre. For example, as shown in Figure 16a, Li et al. [191] proposed rGO/PVA nanocomposites with 80 wt% rGO content by coating the PVA molecules onto GO sheets first, followed by water evaporation-induced self-assembly to form a nacre-like lamellar structure. After further reduction in the GO sheets, Young’s modulus, tensile strength, and fracture strain of the resultant paper-like rGO/PVA nanocomposites were 178%, 75.9%, and ~17.1% larger than those of pure GO paper. Furthermore, as shown in Figure 16b, Gong et al. [179] fabricated laminated GO/CMC (carboxymethyl cellulose) composites using the gel-film transformation method. In this case, with an optimized GO content of 90 wt%, accompanied by the intrinsic hydrogen bonds and additionally introduced ionic bonds Mn^2+^, the tensile strength and toughness of rGO/CMC composites were 4 and 7.3 times larger than those of the pure GO papers, respectively.

## 6. Multi-Scale Mechanical Property Optimization of GLMs

The mechanical properties including modulus, fracture strength, fracture strain, and toughness of GLMs were quite different from monolayer graphene. In this section, we summarize the underlying multi-scale mechanisms to modify the mechanical properties of graphene-based materials to provide the multi-scale optimization routine from nanoscale to macroscale.

The mechanical properties of monolayer graphene are sensitive to various in-plane defects and the morphology of the graphene sheet. For example, defects produce residual stress and stress concentration, which lead to a reduction in strength. Graphene morphologies such as wrinkles and crumpling usually leads to a decrease in Young’s modulus. However, the regulation of defect density and arrangement on grain boundaries can substantially suppress out-of-plane deformation, thus efficiently mitigate the loss of Young’s modulus and strength, while increase the failure strain and fracture toughness. Moreover, mechanisms like dislocation shielding and single-atom chain bridging have been demonstrated to improve fracture toughness.

The mechanical properties of GLNs are mainly affected by the mechanical properties of individual graphene sheets, the sheet size, and interface properties. For nacre-like nanostructure, their tensile mechanical properties can be improved by increasing sheet size and enhancing the interlayer shear properties, while for the laminar nanostructure, their bending rigidity can also be improved by enhancing the interlayer shear properties. Therefore, interlayer properties play an important role in the mechanical properties of both nacre-like and laminar nanostructures. In order to enhance interlayer shear modulus and strength, different types of crosslinks (i.e., covalent, ionic, coordinate, and hydrogen bonds) have been introduced to replace the original weak vdW interactions. Furthermore, interlayer crosslinks also play an important role in the buckling behaviors of graphene laminar nanostructures.

The mechanical properties of graphene-based macroscopic materials including pure graphene assemblies and GO/polymer nanocomposites with high graphene content are strongly affected by their microstructures and nanoscale building blocks. Graphene-based macroscopic materials with well-compacted structures and high-quality graphene sheets were found to possess higher modulus and strength, but less fracture strain than those with wrinkled graphene sheets. Therefore, the additional small molecules introduced as plasticizers can not only smooth wrinkles, but also enhance the interlayer interactions and further enhance the Young’s modulus and strength. For nacre-like GO/polymer nanocomposites, the modulus is comparable to that of pure graphene papers, while toughness and strength have been efficiently enhanced due to the introduction of a polymer as additional interfacial crosslinkers.

The mechanical properties of graphene/polymer nanocomposites depend on the polymer matrix, the graphene nanofillers, and the interface between the two components. In the case of low graphene content, by modifying the interface with chemical crosslinkers, Young’s modulus, strength, toughness, and fracture strain of the composites are comprehensively improved compared to the pure polymers. Furthermore, the continuous fracture mechanism and graphene pre-scaffold method were demonstrated to improve the toughness of the composites.

**Figure 17:**
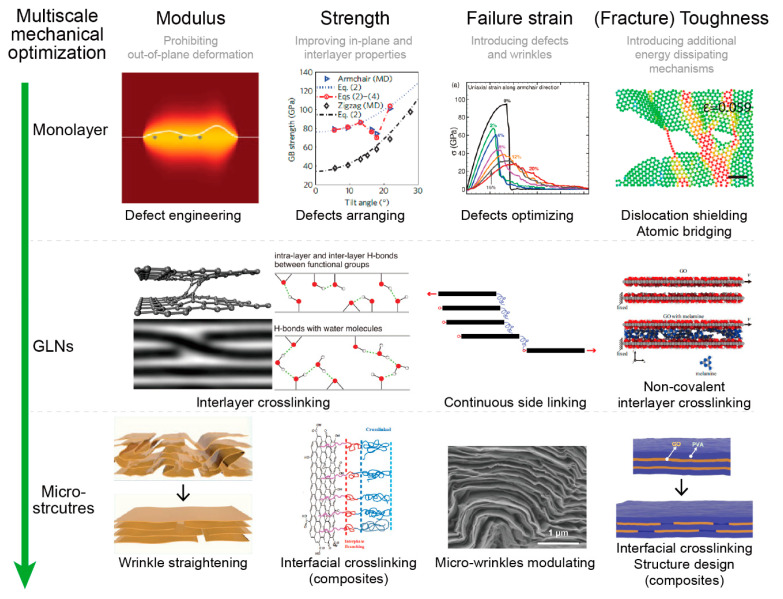
Multi-scale optimization of GLMs. Reprinted with permission from [20,23,26,43,46,67,97,102,113,125,143].

In short, from the nanoscale to macroscale, mechanical properties of graphene-related materials can be improved as follows: (1) improvement of modulus can be obtained by using large-size graphene sheets, enhancing interfacial properties, and straightening the wrinkles; (2) improvement of strength can be obtained by precisely engineering defects (although difficult in experiments) and enhancing the interlayer and interfacial properties; while (3) increase in toughness can be obtained by defect engineering and properly managing the interfacial properties with chemical crosslinkers, as summarized in Figure 17.

## 7. Conclusions and Perspectives

In this review, the multi-scale structures, mechanical behaviors, mechanisms of mechanical degradation as well as the corresponding fabricating methods for graphene-based layered materials are summarized from monolayer graphene to macroscopic graphene assemblies. Multi-scale mechanical optimization strategies have also been proposed based on the mechanical degradation mechanisms. Through our retrospective analysis, we can see that significant efforts have been made to produce the extraordinary properties of monolayer graphene to macroscopic graphene assemblies. However, there is still more space for the improvement in the mechanical properties of graphene-related materials by considering the large gap in the mechanical properties between monolayer graphene and macroscopic graphene assemblies. Here, we suggest a few topics of interest that may help to understand the structure–property relations of graphene-related materials to improve their mechanical properties.

At the nanoscale, works have been conducted to understand the effects of several specific defects on the mechanical properties of monolayer graphene. However, defect type and distribution in real materials may be more complicated (i.e., the effect of curvilinear grain boundaries on mechanical properties has been rarely addressed). Therefore, greater efforts are needed to understand the defect–property relations of 2D materials by machine learning mediated theoretical/numerical methods, based on which defect engineering can be further employed to regulate the mechanical properties. For example, as the Young’s modulus and strength of graphene sheets first decrease and then increase with the increase in grain boundary density, one may obtain the expected mechanical properties by controlling the growth condition of polycrystalline graphene (i.e., temperature) to modulate the grain sizes of graphene in experiments. On the other hand, based on the experimental observations, free-standing monolayer graphene had massive rippling at finite temperatures and the related theory should be developed to consider the rippling effect on mechanical behaviors of monolayer graphene because materials are usually used under finite temperature. In terms of graphene layered nanostructures, in-plane mechanical behaviors of nacre-like structures and bending behaviors of laminar structures are well studied in two-dimensional models. Further mechanical models should be proposed to describe three-dimensional deformation of GLNs as well as the bending behaviors of nacre-like structures. Moreover, both experimental and theoretical works should also be conducted to study the mechanical behaviors of layered structures under finite deformations, which may play important roles in practical applications such as flexible electronics and biological sensors et al.

The mechanical behaviors of macroscopic graphene-related materials are mainly affected by the interfacial properties and microstructures. Previous works have qualitatively explored the effects of interfacial properties, indicating the stronger the interface, the stronger the material. However, more quantitative works involving specific interfacial crosslinkers such as healable and edge crosslinks, interlayer sliding, and the viscoelastic properties of interlayer sliding, are also needed to fully understand the effect of the interface on overall mechanical behaviors. Regarding the microstructures, materials with compact structures are promising for loading bearing, while those with porous structures usually aim toward functional applications such as catalytic et al. As experimentally reported, graphene fibers are usually superior in Young’s modulus and strength to graphene papers. However, the underlying mechanisms are still unclear. Furthermore, the cross-section of GFs usually features core-sheath morphologies, which play important roles on the mechanical behaviors of GFs [192]. Therefore, it is necessary to quantitatively study the geometry–mechanical behavior relation for GFs. An important yet rarely-visited direction is the compressive behaviors of graphene-related materials (except for GAs), which is challenging to address with the current material geometries (fibers, thin films, etc.). Moreover, to better understand these to further modulate the related mechanical properties, general theoretical frameworks should be developed to figure out the multi-scale structure–property relations of macroscopic graphene assemblies. Finally, due to the twisting and bending deformation coupled intriguing properties of graphene, the mechanical–electromagnetic coupled theoretics should also be explored to design graphene-based electrical [193,194] and magnetic [195,196] materials.

## Figures and Tables

**Table 1:** The Young’s modulus and Cauchy ultimate strength of defect-free graphene obtained from different methods.

Method	Potential	Young’s Modulus (TPa)	Armchair Ultimate Tensile Stress (GPa)	Zigzag Ultimate Tensile Stress (GPa)	Ref.
DFT	-	1.050	110	121	[57]
MD	AIREBO	1.01 ± 0.03	102	129	[58]
MD	AIREBO	-	100	126	[59]
MD	Tersoff	1.106	124	130	[60] ^3^
MD	ReaxFF	0.751	125	138	[61]
MM ^1^	Morse	-	102.15	82.22	[62] ^3^
HM ^2^	-	1.030	~118	~104	[63]

^1^ Molecular mechanics; ^2^ Hyperelastic model; ^3^ In terms of engineering stress.

**Table 2:** Summary of up-to-date mechanical properties of GFs and GPs.

Material Type	Graphene Type	Method	Modulus (GPa)	Strength (MPa)	Strain to Failure (%)	Toughness (MJ m^−3^)	Year	Ref.
GFs	GO	Wet-spinning	135	820	-	-	2015	[135]
GFs	rGO	Wet-spinning	400	2200	~0.6	-	2016	[133]
GFs	rGO-ca^2+^	Wet-spinning	-	842.6	3.5	18.5	2016	[136]
GFs	rGO	Dry-spinning	11.6	375	9.4	19.12	2017	[129]
GFs	rGO-ca^2+^	Wet-spinning	-	743.6		26.3	2018	[138]
GB ^1^	rGO	Wet-spinning	309	1900	-	-	2019	[134]
GFs	GO	Twisting film	-	130	29	-	2019	[131]
GFs	rGO	Plasticization Spinning	341.7	3400	-	-	2020	[37]
GPs	rGO-Zn^2+^	Assembling	11.2	439.1	5.3	7.6	2016	[139]
GPs	rGO-AP-DSS	Vacuum filtration	-	538.8	6.6	16.1	2017	[140]
GPs	GO	Sheet collapsing	0.1	23.8	22.7	-	2017	[125]
GPs	dfGO	Thermal annealing	-	-	16	-	2017	[141]
GPs	π-bridged rGO	Vacuum filtration	23.3	1054	6.4	35.8	2019	[142]
GPs	rGO	Plasticization stretching	62.8	1100	-	-	2020	[143]
GPs	rGO	Plasticization stretching	~27	760	-	-	2021	[144]
GPs	rGO	Freeze stretching	65.5	1547	3.7	35.9	2021	[145]

^1^ Graphene belt.

**Table 3:** Summary of up-to-date stiffening, strengthening, and toughening of graphene/polymer composites.

Graphene Structure	Matrix	Filer	Method	Graphene Content	Increment ^1^ of Modulus	Increment of Strength	Increment of Break Elongation	Increment of Toughness	Year	Ref.
Dispersed	PU	PI-GO	Solution mixing	0.1 wt%	47.5%	83.7%	25%	206.5%	2015	[43]
Dispersed	PVDF	PT-g-PMMA-rGO	Solution mixing	0.42 wt%	333%	283%	106%	-	2016	[173]
Dispersed	Epoxy	functionalized graphene	Solution mixing	0.75 wt%	3.6%	4.9%	23.7%	36.3%	2017	[174]
Dispersed	BMI	PTZ-rGO	Solution mixing	0.4 wt%	-	26.5%	-	-	2019	[175]
Dispersed	WPU	Hydroxyl-GO	In situ polymerization	2 wt%	-	139%	15.8%	-	2019	[176]
Dispersed	CNF	PDA-graphene	Vacuum filtration	~10 wt%	-	25.4%	49.8%	-	2020	[177]
Network	Epoxy	Graphene	Freeze casting	0.34 vol%	25.4%	10.2%	-	-	2018	[178]
Network	Epoxy	rGO	Freeze casting	1.24 wt%	-	-14%	-	320% ^2^	2019	[44]
Network	Epoxy	rGO	Freeze casting	0.73 wt%	-	-18%	-	261% ^2^	2020	[45]
Continuous	PVA	GO	Blade coating	0.3 wt%	42.3%	32.2%	64.2%	101.4%	2018	[46]
Nacre-like	rGO	CMC	Gel-film transformation	~90 wt%	-	299.7%	-	633.3%	2017	[179]

^1^ Increment in mechanical properties of the composites compared to those of the pure matrix. ^2^ Increment in fracture toughness.

## Data Availability

Data sharing is not applicable to this article.

## Abbreviations

GO	Graphene oxide
rGO	Reduced graphene oxide
GPs	Graphene papers
GFs	Graphene fibers
GAs	Graphene aerogels
GLNs	Graphene layered nanostructures
GLMs	Graphene-based layer materials
DFT	Density functional theory
MD	Molecule dynamics
GBs	Grain boundaries
DTS	Deformable tension-shear
vdW	van der Waals
